# Burnout in the VHA and the Private Sector

**DOI:** 10.1001/jamanetworkopen.2025.5960

**Published:** 2025-04-01

**Authors:** Eric A. Apaydin

**Affiliations:** Center for the Study of Healthcare Innovation, Implementation and Policy, VA Greater Los Angeles Healthcare System, Los Angeles, California; Department of Medicine, David Geffen School of Medicine, University of California, Los Angeles.

Burnout in health care is a phenomenon that has been studied for decades but has only recently received widespread attention from policymakers, practitioners, and organizations.^1^ This attention accelerated during the COVID-19 pandemic, but high-quality data on the prevalence and causes of burnout were often missing from the public discussion.

## Veterans Health Administration Burnout at Pandemic-Era Peak

Mohr and colleagues^2^ greatly add to this conversation by presenting repeated cross-sectional survey data on burnout from the Veterans Health Administration (VHA) All Employee Survey (AES). The survey of health care workers was administered annually from 2018 to 2023 with sample sizes ranging from 123 271 to 169 448. Their study shows that rates of burnout among health care workers in VHA medical centers were at 30.4% in 2018 and peaked at 39.8% in 2022 before falling to 35.4% in 2023 after the pandemic’s end. Conversely, professional stress mostly declined throughout the pandemic, except for a small increase in 2022, before plummeting in 2023. Full- and part-time telework increased through the pandemic, and burnout and COVID-19–related stress generally declined among those who were able to work virtually. Primary care physicians reported the highest rates of burnout of any occupation, from 45.6% to 57.6%, throughout the study period. Those working in dental, mental health, and rehabilitation service lines also reported large gains in burnout. COVID-19 professional stress decreased the most among nurses, psychologists, audiologists, optometrists, and physical therapists and among those working in emergency medicine, intensive care, administrative areas, and optometry. The report provides many other comparisons among burnout, stress, and factors including geography and facility complexity. Perhaps most importantly, burnout rates are presented descriptively by occupation and service line from 2018 to 2023, as a resource for future work undertaken by researchers, practitioners, and policymakers.

## Need for Organizational-Level Burnout Data

Health care is awash in data these days from claims and electronic health care records, but many organizations do not regularly measure burnout. The National Academy of Medicine, in its recent landmark report on burnout in health care, recommended that burnout be regularly measured by organizations as a crucial first step in understanding and reducing the phenomenon.^1^ Importantly, burnout needs to be measured at an organizational level, as its drivers are embedded within the workplace. The leading theoretical approach to burnout, the job demands-resources model, posits that job demands and resources drive parallel health impairment and motivational process, leading to changes in burnout or work engagement.^3^ The balance of these states of burnout and engagement then lead to improving or worsening job performance. Employing organizational interventions is a key strategy to reduce job demands or increase job resources, ultimately reducing burnout. Before any of this can happen, health care organizations need to regularly measure burnout and track its changes over time.

## Importance of Taking Employee Experience Data Seriously

Workgroup-level AES data are presented to supervisors through internal dashboards after every survey administration, and they are encouraged to share the data with their staff. Data are also aggregated and compared against similar federal employee surveys to rank federal departments as “the best places to work.” The sharing of AES data and its use in changing the workplace are also measured through questions in future surveys. Some VHA AES data are publicly available,^4^ and more detailed data are available to VHA researchers who present a reasonable request to the VHA National Center for Organization Development. Unfortunately, there is no publicly available equivalent of the AES or its burnout data in private sector health care. The American Medical Association has run a series of national surveys on physician burnout using its membership file,^5^ but the data are not public and not representative of any particular health care organization. There is a need for regular, public surveys of burnout among health care workers in private sector health care organizations in the US.

## Leveling the Playing Field

The US government, via the Agency for Healthcare Research and Quality (AHRQ) or the Centers for Medicare & Medicaid Services (CMS), should step in to fill this gap by creating and administering a nationwide survey of health care worker experience, including topics like burnout, well-being, and the working environment. This may seem far-fetched, but Consumer Assessment of Healthcare Providers and Systems (CAHPS), a public, nationwide patient experience survey, was developed for similar reasons.^6^ Before CAHPS, there were many private and public surveys of patient and health plan enrollee experience, but most were not directly comparable or regularly administered. At present, the CAHPS survey has 11 different variations for various health care settings, and the surveys are managed and codeveloped by the AHRQ, CMS, other federal agencies, academic grantees, and contractors. Aggregated CAHPS data are publicly available, but perhaps more importantly, they are used to calculate star ratings for facilities on CMS’ Care Compare website. Patients can use these data, along with other star ratings calculated from quality data, to choose where to receive their care. Health care organizations should receive similar public ratings on worker experience, so that patients and health care workers alike can have the information necessary to choose to receive care or be employed at these institutions.

## Harnessing Worker Experience Data to Reduce Burnout

This new health care worker experience survey could result in health care organizations competing for better working conditions, as clinicians, staff, and patients monitor its results. Patients may not know it, but they are profoundly impacted by the well-being of health care workers, as burnout has been consistently linked to lower quality of care, more medical errors, higher turnover, and worse patient experience. Clinicians and staff, of course, would care about these new surveys and ratings, to avoid workplaces that will result in their burnout. This market-based system for worker experience data already works in the VHA. Leaders are acutely aware of their AES scores, including burnout rates, and they work to make changes to achieve better scores relative to other VHA health care systems or other government agencies. As Mohr and colleagues show,^2^ the VHA has lowered burnout rates since their pandemic peak, but these rates are hard to interpret without equivalent data from non-VHA health care organizations.

## A VHA Initiative to Reduce Burnout

In response to the large increases in burnout reported by health care workers during the pandemic, the VHA recently created the REBOOT (Reduce Employee Burnout and Optimize Organizational Thriving) initiative to develop and implement interventions to reduce burnout.^7^ These interventions include implementing a new chief well-being officer program, reducing the frequency and length of meetings, reducing mandatory online training, and shifting some inpatient nurses to a compressed work week. The VHA may have made these changes anyway, but consistently high burnout rates from the AES were likely a significant motivation. Regularly surveying and publicly reporting health care worker experience, including burnout, may be a good first step toward encouraging private sector health care organizations to take burnout reduction seriously. Burnout may be seen as a fundamental problem in health care in our postpandemic world, but, as always, you cannot change what you do not measure.
